# Global Warming: Arctic Climate: The Heat Is On

**DOI:** 10.1289/ehp.113-a91a

**Published:** 2005-02

**Authors:** David J. Tenenbaum

In November 2004, the Arctic Council, a coordinating body of the eight Arctic nations (Canada, Denmark, Iceland, Sweden, Nor-way, Finland, Russia, and the United States) released its *Arctic Climate Impact Assessment: Impacts of a Warming Arctic* (ACIA). Emerging from the work of hundreds of scientists and incorporating indigenous knowledge about conditions in the Arctic, the ACIA paints a sobering picture of the effect of present and expected global warming on Arctic peoples and ecosystems.

Although a few Arctic areas have cooled over recent decades, overall average surface air temperatures have risen almost twice as much as global averages. In Alaska and western Canada, temperatures have risen 3–4°C since 1954. For the whole Arctic, the ACIA projects additional warming of 4–7°C over the next century. These projections are not a worst-case scenario; they are based on low-to-middle projections from the United Nations Intergovernmental Panel on Climate Change.

The focus of the ACIA was not temperatures, though, but the ecological, cultural, and economic impacts of warming. A few impacts could be positive: marine transportation should improve as ice retreats from shorelines, and vegetation growth may also accelerate in warmer conditions, perhaps increasing food production. But land transportation has already become more difficult due to melting of permafrost; ice highways used to transport industrial and mining equipment are now available for shorter periods each winter. Ice-free oceans, combined with melting permafrost, have already resulted in severe erosion and coastal destruction in Alaska.

In terms of ecology, the impacts are largely negative. Over the past few decades, the amount of boreal forest that burned each year has doubled, and the ACIA says forest insect outbreaks and forest fires are “very likely” to intensify. As ice continues to retreat from the Arctic Ocean, animals that live on or hunt under the ice, including polar bears and several seal species, could grow scarcer or even go extinct.

Caribou in the giant Porcupine River herd in northwest Canada and Alaska are drowning as they cross rivers that normally are still ice when the animals migrate. Warming has also interfered with caribou feeding, says Craig Fleener, a wildlife biologist for the Gwich’in Council International, an indigenous group for whom the Porcupine River herd provides a material and cultural foundation. Repeated freezing and thawing, he says, creates a very hard, crusty layer on the ground that makes it hard for the caribou to reach the lichen that is their major food source.

“What is really happening is rapid climate and environmental change that goes beyond our capability of adapting quickly enough,” says Fleener. “If the change happens over a long period, we can find new methods, a new place to live or harvest, but when you have rapid change, it is much more difficult [to adapt].”

Hunters are also being affected more directly, says Sheila Watt-Cloutier, chair of the Inuit Circumpolar Conference, one of six indigenous organizations that contributed to the report. “As a result of warming,” she says, “Inuit hunters are falling through the thinning ice.” Hunting is the basis of Inuit culture, she stresses, and an economic necessity because imported food prices reflect high transportation costs. The Inuit include 155,000 Arctic natives living in eastern Siberia, North America, and Greenland.

The report is a wake-up call, says Jonathan Foley, director of the Center for Sustainability and the Global Environment at the University of Wisconsin–Madison, but not just for Arctic residents. Polar snow and ice reflect solar radiation out to space, he points out. As the snow and ice retreat, the Earth’s surface gets darker and absorbs more heat, warming the whole planet in a dangerous feedback loop where warming causes melting and melting causes warming.

Watt-Cloutier observes that Arctic peoples already face the threat of high levels of organic pollutants such as dioxin and poly-chlorinated biphenyls that have drifted north through the atmosphere and now contaminate animal fat and human breast milk. With the prospect of greenhouse gases produced further south causing disproportionate warming in the Arctic, the region is once again “being poisoned from afar,” she says.

Arctic people are resilient by necessity and tradition, says Watt-Cloutier, and they have come through enormous, sudden, tumultuous change in the last 50–60 years caused by the introduction of culture, technology, and foods from the temperate regions. But the Arctic way of life is wedded to a specific environment, and that environment is rapidly changing. While much of the press coverage of the ACIA report has focused on the possibility that polar bears could become extinct, she warns, “It’s not just about endangered species of animals. We ourselves are an endangered species.”

